# Comprehensive analysis of aberrant alternative splicing and RNA binding proteins associated with age-related sensorineural hearing loss

**DOI:** 10.1038/s41598-025-20843-8

**Published:** 2025-10-22

**Authors:** Wei Yu, Zeping Qin, XinYu Liang, Xianbai Zhu, Guojing Tan, Baiyang Ren, Yanghong Xiang, Can Zou, Xueqin Zhou, Hongyang Wang, Anchun Deng

**Affiliations:** 1 Department of Otolaryngology, Head and Neck Surgery, Second Affiliated Hospital of Army Medical University, #183 Xinqiao Street, Shapingba District, Chongqing, 637000 People’s Republic of China; 2 Department of Otolaryngology, Head and Neck Surgery, Chenjiaqiao Central Hospital, No. 17 Chendong Road, Shapingba District, Chongqing, 401331 People’s Republic of China; 3 Department of Otolaryngology, Head and Neck Surgery, General Hospital of the People’s Liberation Army, People’s Republic of China (PRC), 28 Fuxing Road, Haidian District, Beijing, 100048 People’s Republic of China

**Keywords:** Age-related sensorineural hearing loss, Alternative splicing regulation, RNA-binding protein, ISG15, UAP1, Computational biology and bioinformatics, Molecular biology

## Abstract

The prevalence of RNA binding proteins (RBPs) in regulating alternative splicing (AS) in age-related sensorineural hearing loss (SNHL) is unclear. To address this question, we comprehensively analyzing RNA-seq data from a mouse model to identify deregulated AS events (RASEs) and differentially expressed RNA binding proteins (DERBPs) associated with age-related SNHL, and constructed the networks between these two levels of changes. We have identified 198 and 187 RASEs related to severe and mild symptoms, respectively. Genes harboring these RASEs are significantly enriched in positive regulation of GTPase activity and cytoskeleton organization. We further identified 25 and 14 DERBPs related to severe and mild symptoms, respectively, and constructed the regulatory network between DERBPs and RASEs. Among these DERBPs, the significantly increased expression of Isg15 and Myom1, the decreased expression of Acan, as well as some of their regulated RASEs including two in Uap1 also demonstrated in cochlear transcriptomes obtained from the age-related SNHL mouse model constructed in this study. Analysis of human pluripotent stem cell-derived macrophages containing ISG15 knockout samples revealed that ISG15 is a key splicing regulator, and can directly regulate the alternative splicing of UAP1 in human cells. These findings prove insights into the involvement of a large number of alternative splicing events driven by RNA-binding proteins (RBPs) in the pathogenesis of age-related SNHL, and suggest their potentials as therapeutic targets and disease markers.

**Supplementary Information:**

The online version contains supplementary material available at 10.1038/s41598-025-20843-8.

## Introduction

Sensorineural hearing loss (SNHL) is a type of hearing impairment resulting from structural and functional damage to the inner ear, vestibular and cochlear nerves^1^. The cochlear hair cells (HCs), vascular wires (SVs) and spiral ganglion neurons (SGNs) are the primary structures affected. SNHL accounts for approximately 90% of all cases of hearing loss, with a prevalence of 27 to 100 per 100,000 individuals per year^2,3^. Globally, more than 50% of individuals aged 60 are affected by age-related SNHL^4^. Different types of sensorineural hearing loss (SNHL) exhibit shared biological mechanisms, such as regulation of apoptosis in cochlea^5,6^, and influence of the survival of SNHL-affected inner ear hair cells and ganglion neurons by autophagy^7,8^. The progressive loss of hair cells and neurons is a key factor in the development of the disease^9,10^^.^. The protection, regeneration, or inhibition of the apoptosis of these cells may represent a therapeutic avenue for SNHL^11–13^. However, the mechanisms underlying the onset and progression of age-related SNHL remain poorly understood.

Alternative splicing represents a distinctive phenomenon among eukaryotes, with a strong correlation with disease^14^. Approximately one-third of disease-causing mutations are associated with aberrant RNA splicing^15^Defective splicing, including alternative splicing defects, splice site mutations, and incorrect splicing, can give rise to novel isoforms and are intimately linked to the progression of SNHL^16–18^. Rohacek et al. demonstrated that the deletion of Epithelial Splicing Regulatory Protein 1 (ESRP1) results in the impairment of gene expression and splicing in the context of cochlear development and auditory function, and mutations in ESRP1 can lead to SNHL^19^. The splicing of a common SLC26A4 mutant associated with SNHL was found to be rescued by antisense oligonucleotides^16^. Additionally, Palma et al. discovered that mutations in calreticulin 23 (Cadherin 23, Cdh23) disrupted the organization of static cilia on hair cells, leading to deafness and vestibular dysfunction in mice. Alternative splicing of Cdh23 was associated with age-related hearing loss in mice^20,21^. Additionally, RNA binding protein Rbm24 is a key factor in regulating inner ear-specific AS and maintaining the auditory and motor coordination functions of the inner ear, partially by directly modulating the effect of Cdh23 splicing on hearing^22,23^. Alternative splicing (AS) is primarily regulated by RNA binding proteins (RBPs), which also play a pivotal role in the pathogenesis of SNHL^24,25^. Despite the above mentioned individual findings, a comprehensive understanding of RBP regulation of AS in age-related SNHL is still lacking. It is reasonable to propose that a large number of alternative splicing events would be changed during the pathogenesis of age-related sensorineural hearing loss (SNHL). Concurrently, the aberrant expression of RBPs in age-related SNHL may serve as a mediator to drive the aberrant alternative splicing of disease-related genes, thereby influencing the development of SNHL.

In this study, we analyzed the RNA-seq data from cochlear tissue derived from an age-related mouse model of sensorineural hearing loss (SNHL)^26^ in order to gain insights into the mechanisms of disease-related alternative splicing driven by RNA-binding proteins (RBPs), and to identify potential therapeutic targets and disease markers. Our findings revealed substantial alterations in alternative splicing in age-related SNHL and its potential regulatory functions, as well as dozens of RBPs potentially mediating these alterations. We have also validate some of the findings by obtaining another set of RNA-seq data from an age-related SNHL mouse model constructed in this study. To our knowledge, this is the first study to delineate the involvement of RBPs in age-associated SNHL using multiple RNA-seq datasets.

## Results

### Age-regulated alternative splicing is prevalent in the age-related SNHL mice

**Fig. 1:**
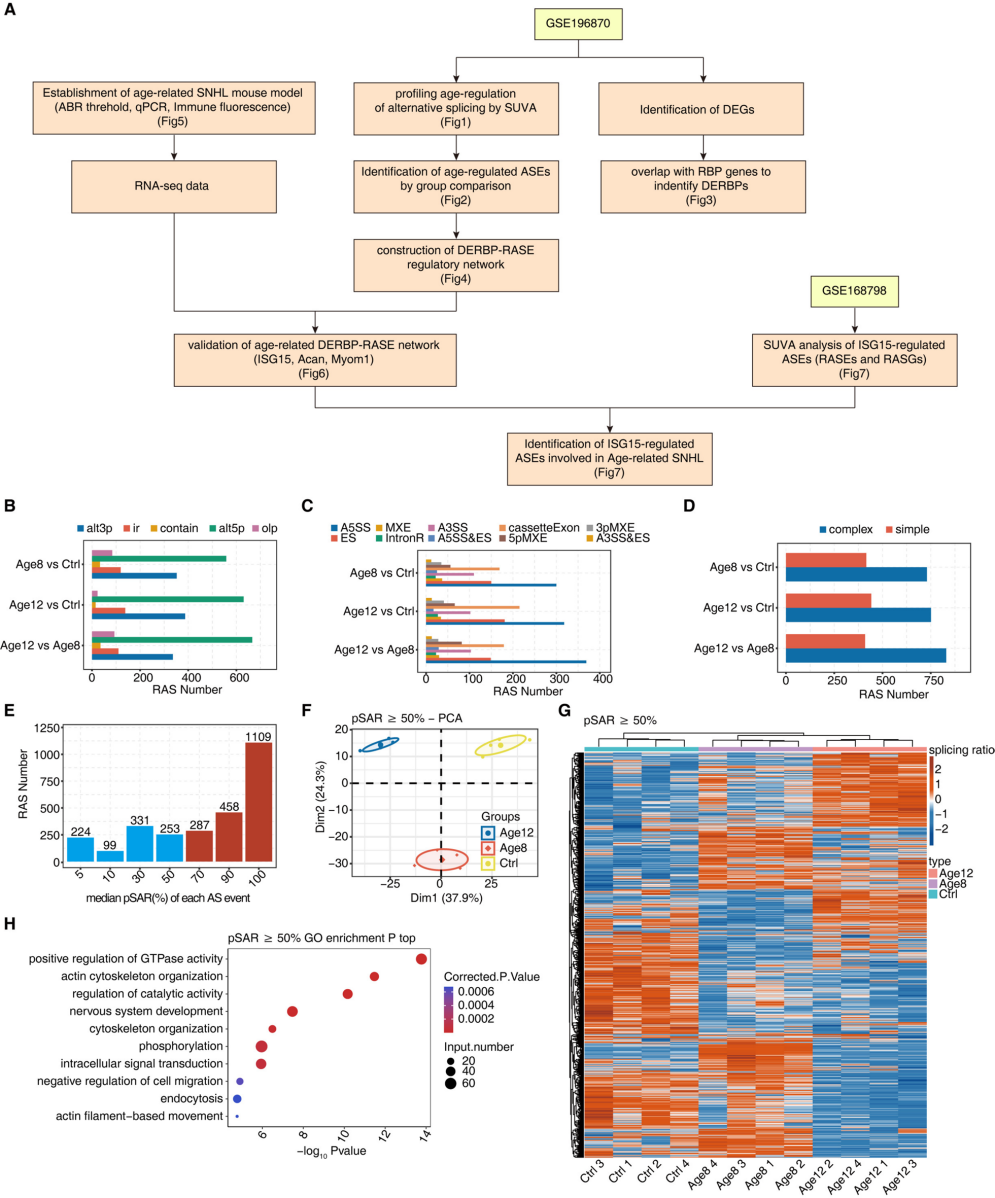
Age-regulated alternative splicing in the age-related SNHL mice. (**A**). Flowchart illustrating the datasets, data analysis and validation strategies of this study. (**B**) Five types of RAS events (RASEs) from three comparisons identified using SUVA. (**C**) Corresponding SUVA-identified RASEs to classical alternative splicing events, A5SS, cassetteExon, ES, A3SS are the most occurring alternative alternative splicing events. (**D**) Bar graph demonstrating RASE with complex splicing and simple plicing. (**E**) Barplot of RASE numbers according to their pSAR value, and1,854 RASEs with pSAR > = 50% were selected for downstream analysis. (**F**) Principal component analysis (PCA) using their splice ratio values using 1,854 RASEs with pSAR > = 50%. (**G**) Heatmap showing RASEs with pSAR > = 50% in samples from Ctrl, Age8, and Age12 groups based on the difference in splice ratio values. (**H**) The scatter plot of GO pathways enriched by RASGs containing 1852 RASEs with pSAR > = 50% in samples.

Chen et al. has recently published three sets of RNA-seq data from the cochleae of three different SNHL mouse models, including one set from age-related SNHL mice (GSE196870 in Fig. 1A). In this model, the hearing capability of 2-month-old normal mice (Ctrl), 8-month-old mice (Age8), and 12-month-old mice (Age12) was measured, which was significantly reduced in Age8 mice and further reduced in Age12 mice^26^. By using SUVA method^27^, we identified regulated alternative splicing events (RASE) that were differentially spliced between Age8 vs Age2, Age12 vs Age2, Age 12 vs Age8 mice (Fig. 1B). Based on splice site usage, SUVA normally characterizes five types of alternative splicing events (ASE), including alternative 3’ splice site (alt3p), alternative 5’ splice site (alt5p), intron retention (ir), a pair of splice sites being contained in another pair of splice site (contain) and a pair of splice sites being overlapped with another pair of splice site (olp). It is shown that the the majority if RASEs were alt5p and alt3p types (Fig. 1B). SUVA classification of ASEs is based on each individual splice site, whereas two pairs of splice sites are involved in defining classical ASEs. Reclassification of SUVA RASEs to the classical alternative splicing events revealed that A5SS (alternative 5’ splice site) was the most frequently occurring RASE, consistent with the SUVA classification (Fig. 1C). The SUVA analysis indicated that a significant proportion of these alternative splicing events were complex splicing events, suggesting that the splicing regulation in cochlear tissue is a sophisticated (Fig. 1D). Furthermore, the Age12 and Age8 groups displayed a more diverse range of differential alternative splicing events compared to the other two comparisons (Fig. 1D), indicating that cochlear alternative splicing in highly regulated by ages and therefore may be related to hearing loss.

We obtained 1854 major splicing events (pSAR > = 50%) for subsequent investigation (Fig. 1E). The principal component analysis of these major splicing events revealed that the Ctrl, Age8, and Age12 groups were significantly separated. Please be noted that the splice ratio of each splice event was used for analysis. This suggests that alternative splicing is deregulated during aging, which represents a critical parameter to distinguish different ages of cochleae (Fig. 1F). The heatmap illustrates the splicing ratio of RAS (pSAR ≥ 50%) across three distinct comparison groups, indicating the presence of a significant fraction of Age12-specifc RASEs which were either upregulated or downregulated in Age12 mice. There are also RASE shared by Age8 and Age12 mice (Fig. 1G). The functional enrichment of genes containing major splicing events were shown in Fig. 1H.

Because aging is a major factor in hearing loss, we proposed that the RASEs between the older cochleae (Age8 and Age12) and the young control (Age2) may be involved in the pathogenesis of age-related sensorineural hearing loss. To test this hypothesis, we performed GO function enrichment analysis. The results showed that the genes containing these age-regulated RASGs are predominantly enriched in the positive regulation of GTPase activity and actin cytoskeleton organization, and both of which play important roles in hearing^28^. We noticed that age-regulated RASGs were also significantly enriched in nervous system development, which further underscore the importance of age-regulated alternative splicing in the pathogenesis of age-related SNHL.

### Identification of age-regulated alternative splicing events (RASE) and genes (RASGs) associated with age-related sensorineural hearing loss

**Fig. 2:**
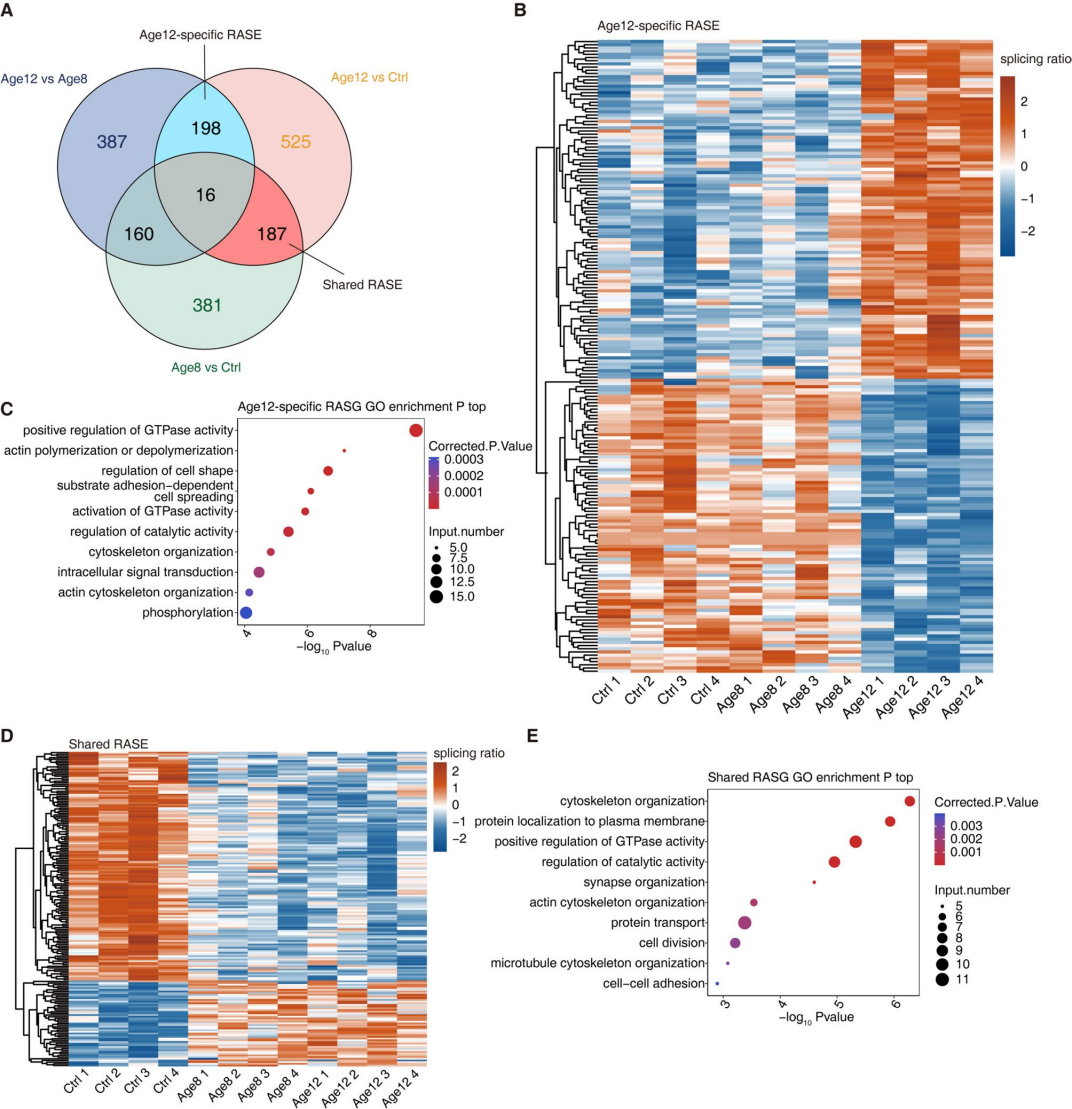
Identification of age-regulated alternative splicing events and genes associated with age-related SNHL. (**A**) Venn diagram showing all RASE among Age12 vs Age8, Age12 vs Ctrl, and Age8 vs Ctrl samples. Age12-specific RASEs indicate those shared by both Age12 vs Age8 and Age12 vs Ctrl comparison, but not by the Age8 vs Ctrl comparison. Shared RASEs indicate those shared by Age12 vs Ctrl and Age8 vs Ctrl comparison, but not by Age12 vs Age8. (**B**) Heatmap of the splice ratio of all Age12-specific RASEs. (**C**) The scatter plot of Age12-specific RASGs enrichment of GO pathways. (**D**) Heatmap of the splice ratio of Shared RASEs. (**E**) The scatter plot of Shared RASGs enrichment of GO pathways.

To further study the involvement of age-regulated alternative splicing events in the pathogenesis of age-related SHNL, we focused on two classes of RASEs. One class is composed of those shared by Age8 vs control and Age12 vs control, which is called Shared RASEs hereafter (Fig. 2A). Given that both Age8 and Age12 displayed hearing loss symptoms, though that of Age12 is more severe^26^, we rationalized that the Shared RASEs should be more related to the mild hearing loss. We defined the RASEs present in both the Age12 vs Age8 and Age12 vs Ctrl comparison groups as the Age12-specific RASEs, which should be more related to severe hearing loss (Fig. 2A). A total of 198 Age12-specific RASEs and 187 Shared RASEs were identified (Fig. 2A). The heatmap illustrates a clear discrepancy of the splicing ratio of Age12-specific RASEs between the Age12 group and the Ctrl and Age8 groups (Fig. 2B). When genes containing the Age12-specific RASEs were subjected to GO functional clustering analysis, it was found that genes containing these Age12-specific RASEs were mainly enriched in positive regulation of GTPase activity, actin polymerization or depolymerization, regulation of cell shape, and matrix adhesion-dependent cell spreading (Fig. 2C). The dynamics of the splice ratio of the Shared RASEs were also plotted, demonstrating a sharp discrepancy between the control group and the Age8 and Age12 groups (Fig. 2D). Genes containing Shared RASEs were enriched in GO biological pathways including cytoskeletal composition, positive regulation of GTPase activity (Fig. 2E). The KEGG pathways enriched by the Age12-specific and Shared RASGs were analyzed and presented in Figure S1, revealing the presence of additional hearing-related function axon guidance^29^.

### Identification of RNA-binding proteins associated with age-related sensorineural hearing loss

**Fig. 3:**
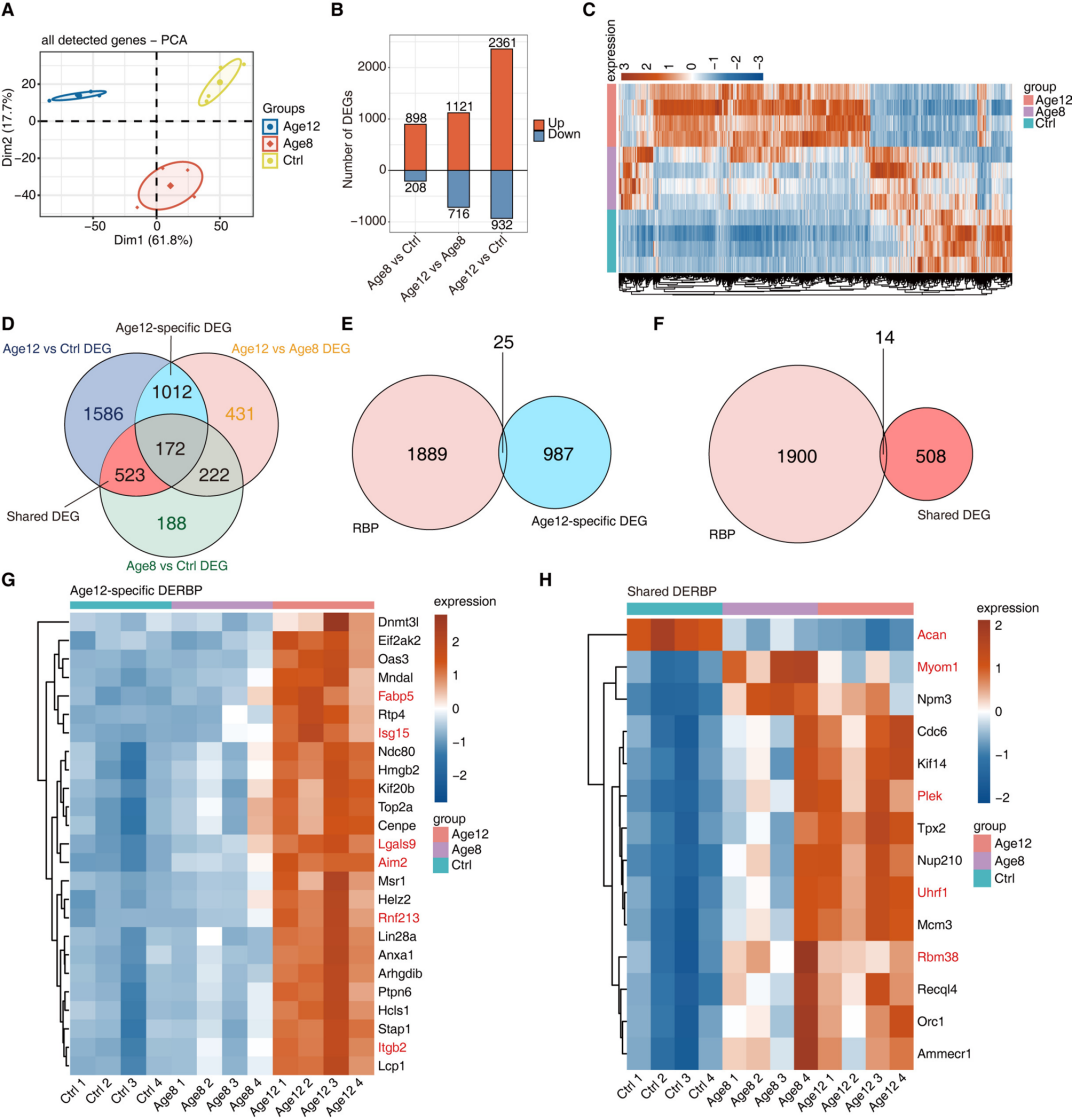
Identification of RNA-binding proteins associated with age-related sensorineural hearing loss. (**A**) Principal component analysis (PCA) using the expression of all detected genes as input. (**B**) The barplot illustrates the quantity of up-regulated and down-regulated differentially expressed genes (DEGs) within the comparison groups of Age8 vs Ctrl, Age12 vs Ctrl, and Age12 vs Age8. (**C**) Heatmap of differential expression of all DEGs in Ctrl, Age8, and Age12 groups. (**D**) Venn diagram showing all DEGs among Age12 vs Age8, Age12 vs Ctrl, and Age8 vs Ctrl comparisons. The definition of Age12-specific DEGs and Shared DEGs were similar to those for RASEs (Fig. 2A). (**E**) Venn diagrams demonstrating the overlap between RBP and Age12-specific DEGs, yielding Age12-specific DERBPs. (**F**) Venn diagrams demonstrating the overlap between RBP and Shared DEGs, yielding Shared DERBPs. (**G**) The heatmap diagram display the expression profile of Age12-specific DERBPs in three comparison groups. (**H**) The heatmap diagram illustrates the expression profile of the Shared DERBPs in three comparative groups.

We next aimed to identify RNA binding proteins that may mediate the age-regulated alternative splicing changes. We proposed that the expression of these candidate splicing regulators display an age-regulated expression pattern. To this end, Principal component analysis of the samples using the expression level of all genes was carried out, showing that the Ctrl, Age8, and Age12 groups were distinctly separated similar as that of alternative splicing change pattern (Fig. 3A, Fig. 1E). Analysis of the differentially expressed genes (DEGs) in three different comparisons showed that the comparison between Age12 and the control group exhibited the largest number of DEGs (Fig. 3B). Heatmap plot of the expression level of all DEGs substantiated this finding (Fig. 3C). We further identified 1012 genes Age12-specific DEGs, which were differentially expressed in both Agr12 vs Age8 and Age12 vs Ctrl but not in the Age8 vs Ctrl group. Additionally, we identified 523 Shared DEGs that were differentially expressed in both Agr12 vs Ctrl and Age8 vs Ctrl but not in the Age12 vs Age8 group (Fig. 3D). Among them, 25 Age12-specific DEGs and 14 Shared DEGs were RNA binding proteins, which were referred as Age12-specific DERBPs and Shared DERBPs, respectively (Fig. 3E-F). The heatmap plots of the expression dynamics of these DERBPs demonstrated that the expression of all but one (Acan) of these 39 DERBPs was increased with ages (Fig. 3G-H). As expected, the age-dependent increase in the expression of Shared DERBPs were pronounced, i.e. a significant increase in Age8 mice, and further significant increase in Age12. One exception is Acan, whose expression is age-dependently decreased (Fig. 3G-H). These findings suggested that the expression of RNA binding proteins in mouse cochleae is primarily induced by aging. It is conceivable that this age-regulated DERBPs plays a critical role in regulating post-transcriptional events such as alternative splicing, and thusly impact on the pathogenesis of age-related sensorineural hearing loss. Consistent with this hypothesis, a number of these DERBPs have been reported to be strongly associated with hearing loss. For example, down-regulated expression of Acan was observed in the auditory cortex of noise-exposed rats when compared to vehicle rats^30^.

### Construction of a co-expression network of RASEs and DERBPs in age-related SNHL

**Fig. 4:**
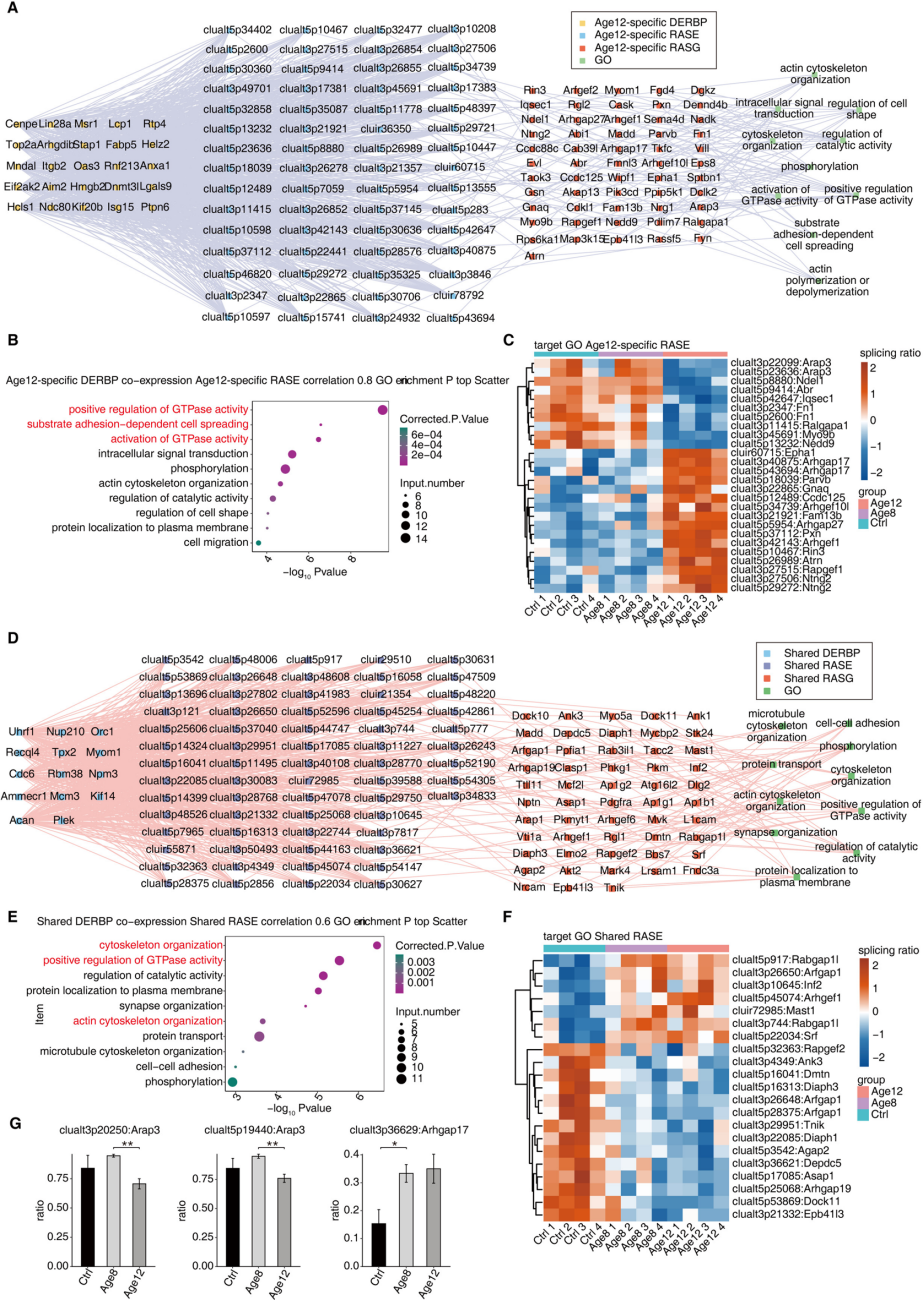
Construction of co-expression networks between RASEs and DERBPs in age-related SNHL. (**A**) Correlation network diagram of Age12-specific DERBP and Age12-specific RASE. Cutoffs of a P value of ≤ 0.01 and a Pearson coefficient of ≥ 0.8 or ≤ −0.8 were used to identify the pairs of co-expression. The corresponding co-expressed GO pathway was also shown. (**B**) Bubble diagram of GO pathways enriched for Age12-specific RASGs containing RASEs co-expressed with Age12-specific DERBP. (**C**) The heatmap diagram showing the splice ratio of Age12-specific RASE. (**D**) Co-expression analysis of Shared DERBP and Shared RASE. Cutoffs of P value ≤ 0.01 and Pearson coefficient ≥ 0.6 or ≤ −0.6 were utilised to recognize the co-expression pairs. The corresponding co-expressed GO pathway was also shown. (**E**) The scatter plot showing the mostly enriched GO biological process resulted from Shared RASGs containing RASEs co-expressed by Shared DERBP. (**F**) The heatmap diagram showing the splice ratio of Shared RASE. (**G**) The splice ratio of three DERBP-correlated RASEs: alt3p20250:Arap3 and clualt5p19440:Arap3 (**C**), clualt3p36629:Arhgap17 (**F**). The statistic significance of the difference was shown. One asterisk* indicates p < 0.05, two asterisks** P < 0.01, and three asterisks*** P < 0.001.

Given the essential roles of RBPs in regulating alternative splicing, the deregulated expression of RBPs is expected to cause the deregulated alternative splicing in the context of age-related SNHL. To identify RASEs potentially regulated by DERBPs, we constructed DERBP-RASE networks based on the correlation between the expression change of Age12-specific and Shared DERBPs and the splice ratio of Age12-specific and Shared RASEs, respectively. The correlated RASEs for Age12-specific and Shared DERBPs were shown (Fig. 4A and D). The functional enrichment analysis was performed for RASGs containing these RASEs (Fig. 4B and E). Age12-specific DERBP-RASGs were significantly enriched in biological pathways including the positive regulation of GTP, substrate adhesion-dependent cell spreading, and activation of GTPase activity (Figs. 4B). Shared DERBP-RASGs were enriched in cytoskeletal formation, positive regulation of GTPase activity, actin cytoskeletal formation and synapse organization (Fig. 4E). The heatmap plots of the splice ratio of these DERBP-correlated RASEs demonstrated the expected dynamics in three different ages of mouse cochleae (Fig. 4C and F). The age-dependent change in the splice ratio of two Age12-specific DEBP-correlated RASEs (clualt5p19440:Arap3 and clualt3p22099:Arap3) and one Shared DEBP-correlated RASEs (clualt3p40875:Arhgap17) were shown in Fig. 4G.

We noticed the previously reported hearing loss-related RBPs Caprin1 and Esrp1 were not identified as a hearing loss-related DERBP. Their expression change showed no observable change (Supplementary Figure S2A). Meanwhile, alternative splicing of some major splicing events of Cdh23 and Slc26a4 were moderately changed in the aged cochlea. The changes of two ASEs of Slc26a4 were at significant levels (Figure S2B-C).

### Establishing a mouse model of age-related SNHL

**Fig. 5:**
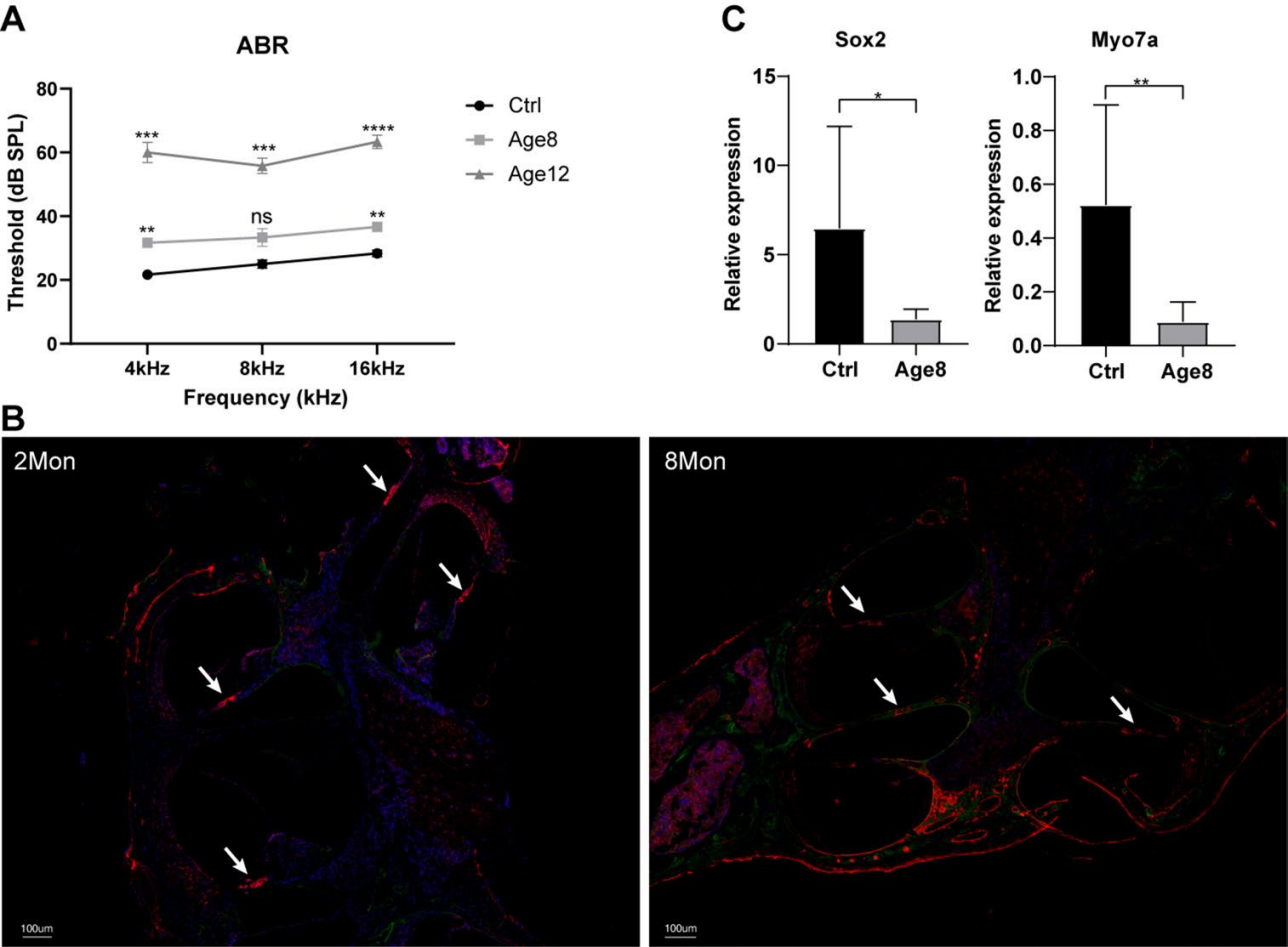
Constructing a mouse model of age-related SNHL. (**A**) ABR thresholds. Six mice were included in each group for the ABR threshold test. Pure tones with different frequencies (4, 8, and 16 Hz) were used as the stimulation sound. The ABR threshold was obtained and plotted. Two asterisk** indicate p < 0.05, three asterisks*** P < 0.01, four asterisks**** P < 0.001. (**B**) Representative immunofluorescence images from whole-mount cochleae using antibodies against Myo7A and Sox2. The ears of 8-month mice exhibited substantial loss of hair cells compared with 2-month cochela. Scale bars, 100 µm. (**C**) RT-qPCR results showing the mRNA level of Myo7A and Sox2 in 2- and 8-month cochela. One asterisk* indicates p < 0.05, two asterisks** P < 0.01.

To validate the Age12-specific and Shared RASEs and DERBPs, as well as their correlation networks, identified in the published dataset, we established an age-related SNHL mouse model by measuring ABR threshold. It is demonstrated the threshold was increased with age, and the increase in Age12 is much more pronounced than in Age8 (Fig. 5A), which is similar as those measured by other methods applied in the previous study^26^. The immunofluorescence and RT-qPCR experiments were performed to detect the expression of Myo7A (marker gene of hair cells) and Sox2 (marker gene of supporting cells) in the cochela of 2-months and 8 month mice. Consistent with the age-related hearing loss phenotype, Myo7A-marked hair cells were diminished, and Sox2-marked supporting cells were relocated in the 8-month cochela compared to those of the 2-month (Fig. 5B). The mRNA levels of both genes were decreased in the 8-month cochela as well (Fig. 5B). These results indicate that the mouse model of age-related SNHL was successful. The mouse cochlear tissue was then taken for transcriptome sequencing, and the data was analyzed and presented below.

### Validation of the DERBP-RASE network using the RNA-seq data obtained from the mouse model established in this study

**Fig. 6:**
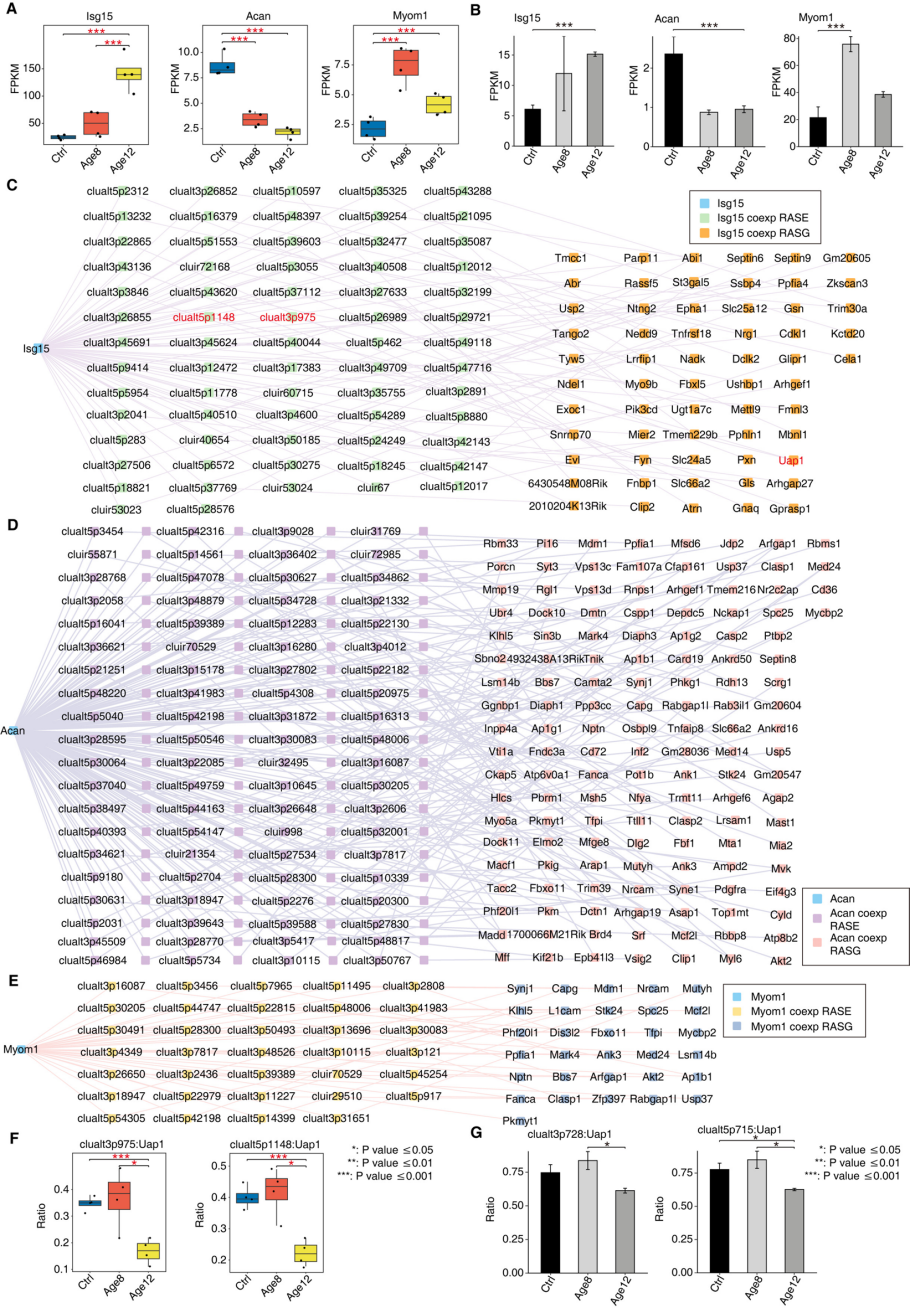
Validation of the DERBP-RASE network. (**A**) Expression of Isg15, Acan, Myom1 in GSE196870. (**B**) expression of Isg15, Acan, and Myom1 in RNA-seq obtained in this study. (**C**) AS network diagram of Isg15 co-expression in GSE196870. (**D**) AS network diagram of Acan co-expression in GSE196870. (**E**) AS network diagram of Myom1 co-expression in GSE196870. (**F**) Splice Ratio of clualt3p975, clualt5p1148 in GSE196870. (**G**) Ratio values of clualt3p728, clualt5p715 in this sutdy RNA-seq. The statistic significance of the difference was shown. One asterisk* indicates p < 0.05, two asterisks** P < 0.01, and three two asterisks*** P < 0.001.

We found that the age-dependent expression patterns of three DERBPs Isg15, Acan and Myom1 were highly consistent in the published RNA-seq data GSE196870 and the RNA-seq data obtained in this study. Isg15 displayed an Age12-specific increase pattern. Acan and Myom1 showed a Shared decrease and increase pattern, respectively (Fig. 6A-B). We noticed that these three RBPs have been previously reported to be involved in hearing loss^28,31,32^, which will be further discussed later. We constructed DERBP-RASE regulatory network specifically for these RNA binding proteins (Fig. 6C-E). We found that a number of these correlated RASEs were replicated in the RNA-seq data generated in this study, two of them from Uap1 were shown (Fig. 6F-G). Please be noted that the alternative splicing of UAP1 has been previously reported to be targets of other RNA binding proteins involving in hearing loss^33^.

We noticed the previously reported hearing loss-related RBPs Caprin1, Esrp1 were not identified as DERBPs from the dataset GSE196870. We analyzed their expression change in the RNA-seq dataset obtained in this study, showing that Esrp1 expression was significantly down-regulated in the aged mouse cochlea (Supplementary Figure S2A). Meanwhile, alternative splicing of some major splicing events of Cdh23 and Slc26a4 were moderately changed in the aged cochlea, not to a significant level (Figure S2B-C).

### ISG15 is a key splicing factor regulating alternative splicing of genes involved in age-related SNHL

**Fig. 7:**
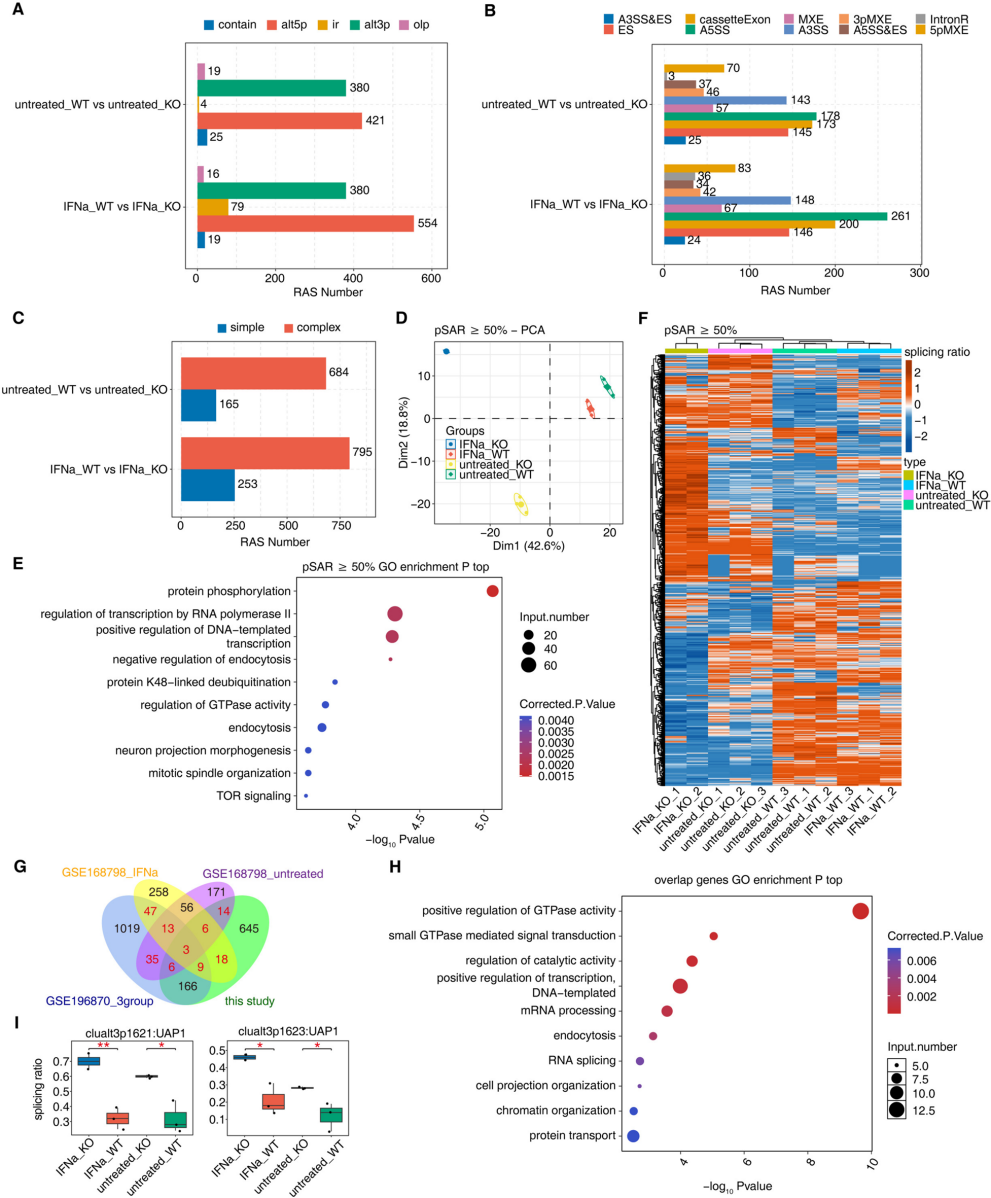
Analysis of ISG15 regulated alternative splicing. (**A**) Five types of RAS events (RASEs) from two comparisons identified using SUVA. (**B**) Corresponding SUVA-identified RASEs to classical alternative splicing events, A5SS, cassetteExon, ES. (**C**) Bar graph demonstrating RASE with complex splicing and simple plicing. (**D**) Principal component analysis (PCA) using their splice ratio values using RASEs with pSAR > = 50%. (**E**) The scatter plot of GO pathways enriched by RASGs containing 1852 RASEs with pSAR > = 50% in samples. (**F**) Heatmap showing RASEs with pSAR > = 50% in all four groups based on the difference in splice ratio values. (**G**) Overlap of RASGs among the untreated and IFNα-stimulated groups of ISG15-regulated alternative splicing genes (GSE168798), as well as the Age12-specific and Shared RASGs identified from GSE196870 and those from the RNA-seq obtained in this study. (**H**) Go enrichment analysis of all the overlapped RASGs between ISG15-regulated alternative splicing genes and Age12-specific and Shared RASGs. (**I**) Plot of the splice ratio of two RASEs of UAP regulated by ISG15 (GSE168798). One asterisk* indicates p < 0.05, two asterisks** P < 0.01.

We noticed that ISG15, Acan and Myom1 are not canonical RNA binding proteins, and their function in regulating alternative splicing has not been reported yet. To further validate the alternative splicing function of DERBPs identified in age-related SNHL mouse model, we searched the public database for RNA-seq datasets containing samples with one of these DERBPs knockout, knockdown or overexpression. We only found one dataset generated from the human pluripotent stem cell-derived macrophages containing ISG15 knockout samples. The wild-type cells and ISG15 knockout cells in the presence or absence of INFα stimulation were used for generating the RNA-seq dataset (GSE168798)^34^. Analysis of this RNA-seq dataset revealed ISG15 regulates thousands of the alternative splicing events in human pluripotent stem cell-derived macrophages (Fig. 7A-B). The majority of the ISG15-regulated alternative splicing events were complex splicing events (Fig. 7C). Principle component analysis using the major splicing events revealed that the alternative splicing profiles of wild-type cells are clustered regardless of INFα treatment, while the ISG15 KO cells are distinctively separated (Fig. 7D), indicating that ISG15 KO substantially changed the alternative splicing profile. This finding was further supported by the heatmap plot of the ISG15-regulated major splicing events (Fig. 7E). Strikingly, we found that biological functions enriched by ISG15-regulated RASGs included regulation of GTPase activity, similar as that found in age-related SNHL mouse model (Fig. 7F). Overlap analysis of the RASGs in three datasets analyzed in this study revealed that 151 ISG-rgulated RASGs were among Shared or Age12-specific RASGs found in age-related SNHL mouse model (Fig. 7G). These genes were mostly enriched in positive regulation of GTPase activity (Fig. 7H). We found that two alternative splicing events of UAP were directly regulated by ISG15 in human macrophage (Fig. 7I). These results together indicate that ISG15 is a powerful alternative splicing regulator, and this molecular activity may explain many of its biological functions including in regulating hearing loss.

## Discussion

By 2025, the global population aged 60 and above will reach 1.2 billion, of which over 500 million will be severely impaired by age-related deafness^35^. Consequently, research on age-related sensorineural hearing loss (SNHL) is of significant scientific and social importance. The present study has revealed over one thousand of alternative splicing events that are age-regulated in the age-related sensorineural hearing loss (SNHL) mice model. Among these age-regulated RASEs in the cochlea, the majority was complex splicing. These findings indicate a large role of alternative splicing regulation, particularly complex alternative splicing, in the pathogenesis of age-related SNHL.

We have identified two groups of RASEs that may be involved in the pathogenesis of age-related SNHL. The Shared RASEs were both changed in Age8 and Age12 mice, which may be involved in a mild symptom. On the other hand, the Age12-specific RASEs were not only changed in Age12 vs control group, but also in Age12 vs Age8 group, indicating a possible function in regulating severe symptom of hearing loss. A total of 198 and 187 of Age12-specific and Shared RASEs was identified in this study, respectively. RASGs containing these disease-related RASEs were predominantly enriched in the pathway of positive regulation of GTPase activity and cytoskeleton organization. Aberrant alternative splicing of SLC26A4 and Cdh23 has been previously linked to hearing loss, and Rbm24 is involved in regulating the hearing related alternative splicing of Cdh23^20–23^. We found that some major splicing events of Slc26a4 and Cdh23 were deregulated in age-related SNHL cochlea, and two of Slc26a4 to the statistical significant levels. These results support a hypothesis that alternative splicing deregulation is prevalent and represents a critical mechnism in the pathogenesis of hearing loss.

Meanwhile, we have also identified 25 and 14 Age12-specific and Shared DERBPs, respectively, which demonstrating the corresponding age-dependent expression profiles. Construction of the expression network between DERBPs-RASEs led to splicing regulation cues between the splicing regulators and their targets.

Some RNA binding proteins have been suggested to play critical role in the pathogenesis of hearing loss. For example, deletion of RBP Caprin1, which regulates the normal state of IHC-SGN synapses, results in progressive hearing loss^24^. Mutations in ESRP1 can lead to SNHL^19^. In this study, we found that the mRNA levels of neither genes in the mouse cochlea were significantly changed by aging. This study revealed 39 DERBPs that may be involved in mild or severe age-related SNHL, including Fabp 5, Lgals9, Aim 2, Rnf213, and Itgb 2, Isg15, Acan, Myom1, Plek, Uhrf1, and Rbm 38, indicating the large role of RBPs in regulating the pathogenesis of hearing loss. Most of these DERBPs were not canonical RNA binding proteins but with suggested RNA binding activity. Our findings suggest their functions in regulating the alternative splicing of GTPase activity and cytoskeleton organization, and this regulation could be linked to the pathogenesis of age-related SNHL, which has not been reported before.

In support of the pathogenesis roles of disease related DERBPs identified in this study, all above mentioned DERBPs have suggest to be involved in regulating hearing loss. FABP5, a lipid-metabolising protein, was upregulated in the inner ear and may play a role in the atherosclerotic lesions of the inner ear^36^. Lgals9 may play a role in macrophage activation within the cochlea of aged mice, and chronic inflammation may be a contributing factor in age-associated cochlear degeneration^37^. Consistent with our findings, Lgals9 expression was elevated in both post-noise cochlear outer hair cells and senescent cochlear hair cells^38^. The accumulation of AIM2 protein in the cochlear SGN was found to be significantly elevated in cases of cytomegalovirus (CMV) infection-induced sensorineural hearing loss^39^. Caseinolytic peptidase (CLPP) null mice have auditory defects, and deletion of CLPP in the mitochondrial matrix induces a strong nuclear response in all tissues, activating inflammatory and immune mediators, which includes up-regulation of Rnf213^40^. Itgb2 SNP is associated with an increased susceptibility to sudden onset SNHL^41^. The cell surface receptor Itgb2, which plays a role in the recruitment and activation of macrophages and microglia, was found to be expressed by cochlear macrophages^42,43^. ISG5 is one of the earliest ISGs (type I Interferons induced gene), whose expression is elevated in a number of different diseases, including inflammatory, traumatic, neoplastic, and degenerative diseases^31,44^. The expression of Isg15 was significantly upregulated in hair cells affected by noise-induced hidden hearing loss^45^. Down-regulated expression of Acan was observed in the auditory cortex of noise-exposed rats when compared to vehicle rats^30^. Furthermore, Myom1 expression was found to be markedly elevated in the cochlea of mice following sound overstimulation^46^. Plek is expressed in the cochlea of the adult mouse, including spiral ganglion neurons, and represents an important intermediate in the pathway for secretion and activation of the pro-inflammatory cytokines TNF-α and IL-1β. Alterations in the expression levels of Plek in the auditory organ may be responsible for the aetiology of DFNA58-type deafness^47^. Uhrf1 has been demonstrated to induce COX26 methylation, thereby exacerbating cochlear damage caused by intermittent hypoxia^48^. Calcineurin 23 (CDH23), which forms part of the tip junction, plays a crucial role in mechanoelectrical transduction (MET) in hair cells. Errors in the process of alternative splicing of this gene can lead to hearing loss. Rbm38 overexpression has been observed to enhance the inclusion of exon 68 of the Cdh23 minigene^49^.

GTPase activity has been widely reported to be involved in hearing loss. For example, traumatic noise induces transient cellular ATP depletion that activates the Rho-GTPase pathway, leading to cochlear outer hair cell death^50^. It is noteworthy that Rho GTPases are essential regulators of the actin cytoskeleton and have been implicated in hearing loss^28,50^. This suggests that our finding of the enrichment of the age-regulated alternative splicing in positive regulation of GTPase activity and cytoskeleton organization could be coordinated by alternative splicing regulation of Rho GTPases. This assumption is supported by the results discussed below.

During the development of cochlear hair cells, Rho GTPases are activated by various extracellular signals to further stimulate multiple downstream effectors. RhoA, Cdc42, and Rac1 are members of the classical subfamily of the Rho GTPase family, critical in the highly polar auditory sensory system, and have been considered as therapeutic drug targets for deafness (ref)^29,50^. Arhgef6 is a member of the family of guanine nucleotide exchange factors (GEFs) of Rho GTPases that specifically activates the Rho GTPases CDC42 and RAC1. Arhgef6 is expressed in mouse hair cells, and its deletion leads to progressive hearing loss. One of the clinical features of patients carrying Arhgef6 mutations is SNHL^51^. Importantly, we have found that alternative splicing of Arhgef6 was among the Shared RASEs, while those of Arhgef1 ad Arhgef10 were among the Age12-specific RASEs identified in age-related SNHL mice. All of them were the predicted targets of DERBPs. To our knowledge, this is the first report of the involvement of the alternative splicing of Arhgef6, Arhgef1 and Arhgef10 in hearing loss. Our finding highlights the importance of alternative splicing of the family of guanine nucleotide exchange factors (GEFs) of Rho GTPases in regulating age-related SNHL, which deserves further investigation in the aspects of pathogenesis and therapeutic application.

lt is noteworthy that alternative splicing of several members of Rho GTPase activating protein family were among the DERBP-regulated RASEs, which included 2 RASEs of Arhgap17, 3 RASEs of Arhgap1, and 1 RASE of Arggap19. Arhgap17, Rho GTPase activating protein 17, belongs to the small G protein family members. The tight junction integrity and cell formation is maintained by Arhgap17 and Amot complex, which is mediated through a coordinated regulatory mechanism of Cdc42^52^. Arhgap17 plays a role in cytoskeleton formation by catalyzing the GTPase activity of Rho family proteins and inducing the reorganization of cortical actin filaments^53^. Our finding that alternative splicing of Arhgap17, Arhgap1 and Arhgap19 is collectively changed during the pathogenesis course of age-related SNHL suggests a critical role of Rho GTPase activating proteins and their alternative splicing regulation in the development of this disease, which could be novel therapeutic targets and require further investigation.

In summary, our findings highlight that the alternative splicing of Rho GTPase and their activator proteins could be a critical mechanism driving the pathogenesis of age-related SNHL, and that prevention of the specific deregulation of the alternative splicing of these GTPase activity could be a novel therapeutic approach against age-related SNHL.

Some other RASGs containing RASEs, potential targets of DERBPs, have also been implicated in encoding hearing capability. Fibronectin (Fn1) is involved in chronic hearing loss^54^. Myosin (Myo9b) is expressed in the inner ear and plays a crucial role in the auditory system^55^. The INF2 gene encodes a formic acid protein that interacts with the Rho-GTPase CDC42 and the myelin and lymphocyte proteins (MAL). Its mutation has been associated with SNHL^56^. An increase in DIAPH3 activity has been observed in patients with auditory neuropathy, which is associated with hearing deficits^57^. Defects in DIAPH1 and DIAPH3 have been linked to various forms of hearing impairment in humans. Heterozygous mutations in DIAPH1 result in autosomal dominant deafness, with or without thrombocytopenia, whereas mutations that induce overexpression of DIAPH3 lead to autosomal dominant hereditary auditory neuropathy type^58^. DIAPH1 mutations have been identified as a cause of autosomal dominant non-syndromic sensorineural hearing loss. The activation of the structurally truncated C-terminal truncation of DIAPH1 can also lead to human sensorineural hearing loss^59^. EPB41L3 is essential for the development and function of hair cells^60^. In conclusion, the RASGs containing disease-related RASEs identified in this study are closely associated with age-related SNHL. Our findings suggest that their alternative splicing could represent a molecular mechanism regulating the pathogenesis of age-related SNHL. Further investigation is worthy to further elucidate the function of these RASEs in this disease.

We have constructed an age-related SNHL mouse model, and RNA-seq data from this model has validated the age-regulated expression of three of the identified DERBPs Isg15, Myom1 and Acan, with Isg15 demonstrating an Age12-specifc increase in expression, while Myom1 and Acan demonstrating an age-dependent increase and decrease in expression, respectively. Consistently, the significantly upregulated expression of Isg15 in hair cells was stimulated by noise-induced hidden hearing loss^61^ and down-regulated expression of Acan in the auditory cortex of noise-exposed rats^30^ has been demonstrated previously, which further validated the findings of this study.

The Isg15 protein is typically not expressed under physiological conditions but is abnormally elevated in multiple pathological diseases, such as in neurodegenerative diseases^62^. Therefore, the Isg15 protein is now being considered a diagnostic/prognostic biomarker and therapeutic target^31^. Specifically, Isg15 was markedly elevated in the brains of mice with global cerebral ischaemia and traumatic brain injury, indicating that Isg15 may serve as a suitable biomarker for the detection of neuronal injury in the central nervous system (CNS)^63^. The upregulation of Isg15 was evident in neuronal tissues and was correlated with the degeneration of sensory neurons in Clec16a knockout mice^64^. Our finding of Age12-specific expression of Isg12 is consistent with the the aforementioned findings, and the co-expression network of Isg15 with RASEs suggest that Isg15 exert some of its function in age-related SNHL via a previously unreported mechanism, i.e. regulation of alternative splicing.

Two RASEs of Uap1 were among the potential RASE targets of Isg15 validated by the RNA-sea data obtained in this study. UAP1 is a protein pyrophosphorylase regulating interferon (IFN) response to protein serine pyrophosphorylation, and an important positive regulator of the type I IFN signaling pathway^65^. The alternative splicing of UAP1 has been previously reported to be targets of other RNA binding proteins involving in hearing loss^66^. This study suggests that alternative splicing of UAP1 is a direct target of ISG15. Taken together, we propose that ISG15 promotes age-related SNHL by acting as splicing factor regulate the alternative splicing of the UAP1, as well as other genes including those of positive regulation of GTPase activity. These findings highlight the critical role of ISG15 in pathogenesis of age-related SNHL, and its potential as a therapeutic target and biomarker.

In summary, this is the inaugural high-throughput data system to analyse the mechanism by which RBP regulates selective splicing in age-related sensorineural hearing loss (SNHL). The findings of our study indicate that alternative splicing (AS) is significantly altered and plays a regulatory role in age-related sensorineural hearing loss (SNHL). Additionally, AS is predominantly enriched in pathways that regulate GTPase activity. Following network co-expression and validation analyses, it was determined that RBP Isg15 may regulate the AS of the Uap1 gene, thereby affecting the mechanism of age-related SNHL. The precise mechanism remains to be elucidated through further investigation.

This study presents the first high-throughput data-driven analysis of RNA-binding protein (RBP)-mediated alternative splicing mechanisms in age-related sensorineural hearing loss (SNHL). Our findings demonstrate the prevalence of alternative splicing dysregulation in positive regulation of GTPase activity and cytoskeleton organization, particularly Rho GTPase and their activator proteins, which may contribute to SNHL pathogenesis. We have demonstrated that RNA binding protein ISG15 is a key alternative splicing regulator which may drive the pathogenesis of age-related SNHLvia modulating alternative splicing of Uap1 AS events and positive regulation of GTPase activity. Therefore ISG15 is a potential therapeutic target in preventing age-related SNHL, which warrants further investigation.

### Limitation of the study

We are aware that this study has several limitation. Firstly, the age-related SNHL model is complicated by the factor of aging, which could not be discriminated from the disease factor of hearing loss. Secondly, the DERBP-RASE network was built by co-expression analysis. We have obtained the ISG15-regulated alternative splicing profile by analyzing ISG15 KO macrophage, further study in hair cells or neuronal cells is warranted. Thirdly, a lack of validation of the major findings in clinical data, which limits their clinical relevance. Given the limitation regarding the DERBP-RASE network built by co-expression analysis (a correlation not causation), targeted DERBP knockdown/overexpression in inner ear cell lines, followed by functional assays and direct splicing analyses are required to further explore the major findings of this study.

## Materials and methods

### Ethics declaration

The use of animals in this study was approved by the Ethics Committee for Laboratory Animal Welfare of the People’s Liberation Army Army Medical University. The ethical number was AMUWEC20245258, and all methods were carried out in accordance with the relevant guidelines and regulations. All methods are reported in accordance with ARRIVE guidelines (https://arriveguidelines.org). The animals used in the experiments were C57BL/6 J mice, aged approximately 2 months, 8 months and 12 months according to the experimental grouping.

### Establishment of SNHL mouse model

Mice were purchased from Chongqing Lapidary Biotechnology Co. Male and female mice were bred and housed under the required specific pathogen free conditions. Mice were divided into 2-month group, 8-month group and 12-month group. Six mice for each experimental group were anaesthetised using sodium pentobarbital by intraperitoneal injection and then the Auditory Brainstem Response (ABR) threshold test was carried out. We used alternating acoustic stimulation in both ears, and the stimulation sound used pure tones with different frequencies (4, 8, and 16 Hz), and the stimulation intensity was started from 90 dB, and decreased in 5 dBd increments until no III waveform could be detected. The lowest intensity that could be measured at each stimulation frequency was recorded, which was the threshold value of the mouse. ANOVA was performed on the hearing thresholds of the three groups of mice obtained, and the differences between groups were statistically significant.

### Tissue preparation and sectioning

Fresh tissues were immediately fixed in 4% paraformaldehyde (PFA) for 24 h at room temperature. Following fixation, tissues were trimmed and placed in embedding cassettes. Dehydration was performed using a graded ethanol series (75%, 85%, 90%, 95%, and 100% ethanol, 30 min each), followed by clearing in xylene (5–10 min) and infiltration with molten paraffin wax (65 °C, 1 h × 3). Tissues were embedded in paraffin blocks, cooled on a −20 °C freezing stage, and sectioned at 4 μm thickness using a microtome. Sections were floated on a 40 °C water bath, mounted onto glass slides, and dried at 60 °C overnight.

### Immunofluorescence staining

Sections were deparaffinized in xylene (10 min × 3) and rehydrated through graded ethanol (100%, 95%, and 70%, 5 min each). Antigen retrieval was performed in citrate buffer (95 °C, 15 min) followed by cooling to room temperature. After washing with PBS (3 × 5 min), sections were encircled with a hydrophobic barrier and blocked with 3% bovine serum albumin (BSA) for 30 min. Primary antibodies (Anti-SOX2 Rabbit pAb, #GB11249, Servicebio; Anti-MYO7A Rabbit mAb, #R382408, Zenbio) were applied and incubated overnight at 4 °C. After PBS washes, fluorescent secondary antibodies were applied for 50 min at room temperature in the dark. Nuclei were counterstained with DAPI (10 min), and tissue autofluorescence was quenched with sodium borohydride (5 min). Sections were washed, mounted with antifade medium, and imaged using a fluorescence microscope (NIKON ECLIPSE C1) (DAPI: 330–380 nm ex/420 nm em; FITC: 465–495 nm ex/515–555 nm em; CY3: 510–560 nm ex/590 nm em; CY5: 608–648 nm ex/672–712 nm em).

### Quantitative real-time PCR

The extracted total RNA was reverse transcribed into cDNA using a HiScript III RT SuperMix kit with gDNA wiper (R323-01, Vazyme, China) at 42˚C for 5 min, 37 ˚C for 15 min, 85 ˚C for 5 s performed on the mycycler(T100, Bio-Rad, USA). The qRT-PCR assays were performed with HieffTM qPCR SYBR® Green Master Mix (Low Rox Plus) (11202ES03, Yeasen, China) by ABI QuantStudio 5, followed by denaturing at 95˚C for 10 min, 40 cycles of denaturing at 95˚C for 15 s and annealing and extension at 60˚C for 1 min. Each sample had three technical replicates. qRT-PCR data were calculated with the 2^- ΔΔCT^ method and GAPDH was used as an endogenous reference control. Comparisons were performed with the two-way ANOVA by using GraphPad Prism software (Version number8.0, San Diego, CA). Primer sequences used in this study were as follows:

Myo7A:

Forward 5’ -ATGGCACAGAGGAATCAAGGA-3’,

Reverse 5’-TGGAGCAGATGATACTGAGGAG-3’;

Sox2:

Forward 5’ -TTGTTCAATCCTACCCTTTC-3’,

Reverse 5’-CTGATTCCAATAACAGAGCC-3’;

Gapdh:

Forward 5’-GGAGATGCTCAGTGTTGG-3’,

Reverse 5’-TGACAATGAATACGGCTACA-3’.

### RNA extraction and obtaining RNA-seq data from mice

After the test was completed, the mice were euthanised using an overdose of sodium pentobarbital, and the cochleae were collected and quickly frozen in dry ice and stored at −80 C until total RNA extraction. The RNA-Seq assay was performed by Wuhan Ruixing Biotechnology Co., Ltd. (http://www.rxbio.cc). For RNA preparation, DNA was removed by treatment with RQ1 DNase (M6101, Promega, USA). RNA-seq library preparation was performed using the VAHTS® Universal V8 RNA-seq Library Preparation Kit (NR605, Vazyme, China) for targeted RNA-seq library preparation. The mRNA was captured with VAHTS mRNA Capture Beads (N401, Vazyme, China). The fragmented mRNA was then converted to cDNA. After end repair and A-tailing, the cDNA was ligated to VAHTS RNA Multiplex Oligos 1 from Lily (N323, Vazyme, China), amplified, purified, quantified before sequencing, and stored at −80 °C. Strands labeled with dUTP (2nd cDNA strand) were not amplified, allowing strand-specific sequencing. For high-throughput sequencing, libraries were prepared according to the manufacturer’s instructions and applied to the DNBSEQ-T7 system for 150 nt pair-end sequencing.

### Retrieval and process of public data

Public sequence data files GSE196870^26^ and GSE168798^34^ were downloaded from the Sequence Read Archive (SRA) (https://www.ncbi.nlm.nih.gov/geo). SRA Run files were converted to fastq format with NCBI SRA Tool fastq-dump (v.2.8.0).

### Process of the public data and sequence data obtained in this study

The raw reads were trimmed of low-quality bases using a FASTX-Toolkit (v.0.0.13; http://hannonlab.cshl.edu/fastx_toolkit/). Then the clean reads were evaluated using FastQC (http://www.bioinformatics.babraham.ac.uk/projects/fastqc).

### Reads alignment and differentially expressed gene (DEG) analysis

The retrieved clean reads from GSE196870 and generated in this study were aligned onto the mouse genome version GRCm39, while those from GSE168798 were aligned onto the human genome version GRCh38_v45 using HISAT2(v.2.2.1)^67^. Uniquely mapped reads were selected for further analysis, and the number of reads located on each gene was calculated. The expression levels of genes were evaluated using FPKM (fragments per kilobase of exon per million fragments mapped). DEseq2 (v.1.30.1) software was used to perform differential gene expression analysis using the reads count file^68^. DEseq2 was also used to analyze the differential expression between two or more samples and thus determine whether a gene was differentially expressed by calculating the fold change (FC) and false discovery rate (FDR), FC ≥ 2 or ≤ 0.5, FDR ≤ 0.05.

### Alternative splicing analysis

Regulatory alternative splicing events (RAS) were defined and quantified using the SUVA (v2.0) pipeline^27^. The differential splicing between two groups of sample in each comparison was analyzed. Proportion of each SUVA AS event reads (pSAR) was calculated. We used pSAR > 50% as the criterion to select the major splicing event among all detected splicing events corresponding to a specific splice site.

### Construction of DERBP–RASE regulatory network

The DERBP–RASE regulatory network was constructed by building the correlation network of the expression change of DERBPs and splicing ratio change of RASEs (pSAR ≥ 50%) in each comparison group. Pearson correlation coefficient was calculated. Age12-specific DERBP and RASE pairs satisfying absolute value of correlation coefficient ≥ 0.8 and P value ≤ 0.01 were selected. Shared DERBP and RASE pairs satisfying absolute value of correlation coefficient ≥ 0.6 and P value ≤ 0.01 were selected.

Co-expression analysis was performed between Age12-specific RAS (pSAR ≥ 50%) and RBPs Isg15, Acan and Myom1, separately. For Isg15, the co-expression threshold was set as an absolute value of correlation coefficient ≥ 0.8 and P value ≤ 0.01, while those for Acan and Myom1 was coefficient ≥ 0.6 and P value ≤ 0.01.

### Identification of differentially expressed RBPs in the groups

Then expression profile of differentially expressed RBPs were filtered out from all DEGs according to a catalog of 2,141 RNA-binding proteins (RBPs) retrieved from four previous reports^69–72^.

### Functional enrichment analysis

Gene Ontology (GO) terms and KEGG pathways were identified using KOBAS 2.0^73–76^. Hypergeometric test and Benjamini–Hochberg FDR controlling procedure were used to define the enrichment of each term.

### Other statistical analysis

Principal component analysis (PCA) analysis was performed by R package factoextra (https://cloud.r-project.org/package=factoextra) to show the clustering of samples according to the first two components. After normalizing the reads by TPM (Tags Per Million) of each gene in samples, in house-script (sogen) was used for visualization of next-generation sequence data and genomic annotations. The pheatmap package (https://cran.r-project.org/web/packages/pheatmap/index.html) in R was used to perform the clustering based on Euclidean distance. Student’s *t*-test was used for comparisons between two groups.

## Supplementary Information

Supplementary Information 1.

Supplementary Information 2.

## Data Availability

The sequence data were deposited into the Gene Expression Omnibus database under accession number GSE297020 and are available at the following URL:https://www.ncbi.nlm.nih.gov/geo/query/acc.cgi?acc=GSE297020. The secure token for reviewer access is: ebkjygegzdgplmf.
