# Supplementary figures and images for: Comprehensive analysis of aberrant alternative splicing and RNA binding proteins associated with age-related sensorineural hearing loss
Source: Sci Rep. 2025 Oct 22;15:36956. doi: 10.1038/s41598-025-20843-8 (PMC12546835; doi:10.1038/s41598-025-20843-8)

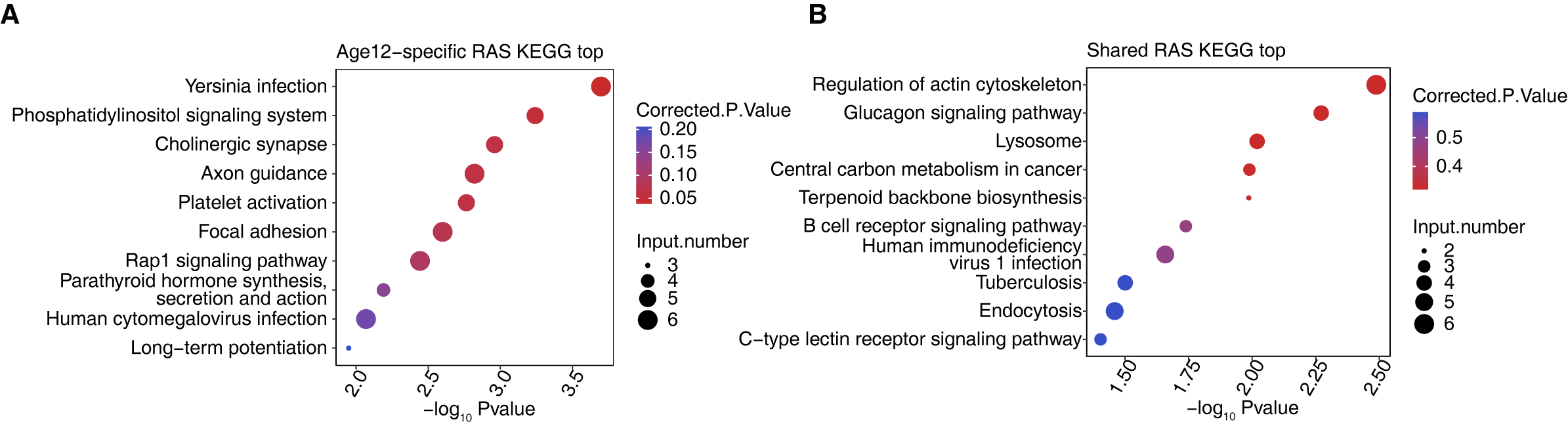

Supplement: Supplementary file 1 — Supplementary Information 1. [file 41598_2025_20843_MOESM1_ESM.tif]

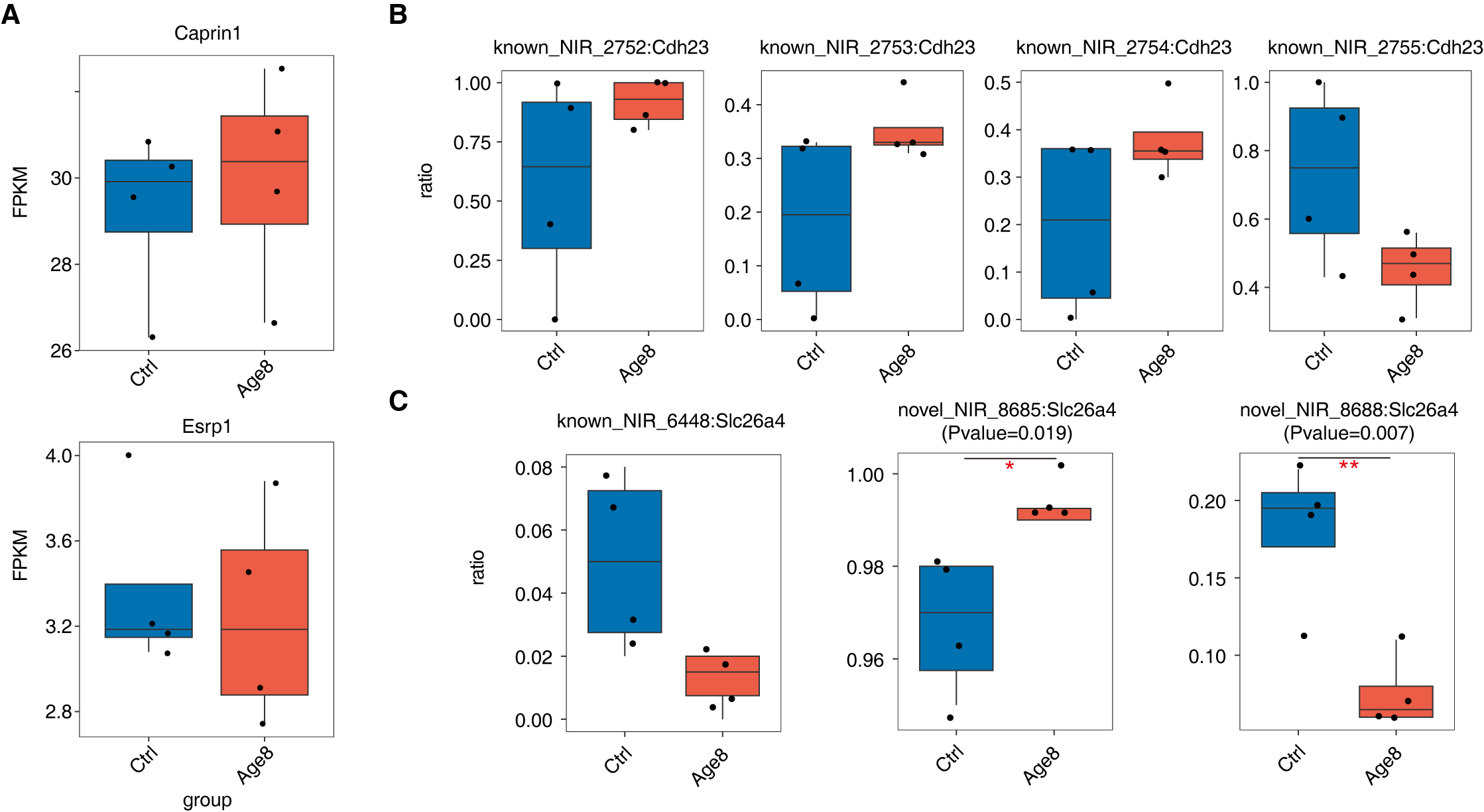

Supplement: Supplementary file 2 — Supplementary Information 2. [file 41598_2025_20843_MOESM2_ESM.tif]
